# Mechanical properties and biocompatibility of graphene-reinforced materials for crowns and bridges: A systematic review and meta-analysis with emphasis on ceramics

**DOI:** 10.1016/j.jobcr.2025.09.021

**Published:** 2025-09-24

**Authors:** Anes Adnan Alshamaa, Ibrahim Hamad Alfahdawi, Mohamed Abdulmunem Abdulateef, Abdulkhaleq Mohammed Qaraghuli

**Affiliations:** a Oral Diagnosis Department, College of Dentistry, University of Al-Maarif, Anbar, Iraq; b College of Dentistry, University of Al-Maarif, Anbar, Iraq; c Department of Medical Biotechnologies, Division of Fixed Prosthodontics, University of Siena, Siena, Italy

**Keywords:** Graphene, Dental ceramics, Mechanical characteristics, Crowns, Bridges, Biocompatibility, Meta-analysis, Systematic review

## Abstract

**Objective:**

This systematic review and meta-analysis investigated the mechanical characteristics and biocompatibility of graphene-reinforced materials, especially ceramics for dental crowns and bridges. Its goal was to synthesize the available evidence and highlight areas needed for future research.

**Methods:**

A systematic search was conducted on PubMed, Web of Science, Science Direct, and Google Scholar, following the PRISMA guidelines. Eight in vitro studies were included which assessed biocompatibility and mechanical performance, such as flexural strength, compressive strength, and hardness. The QUIN tool was used to assess the risk of bias, and random-effects models were used for the meta-analysis.

**Results:**

Graphene reinforcement significantly improved mechanical properties, with flexural strength increasing by ∼100 MPa in some ceramic systems (SMD: 1.26, 95 % CI: −0.20, 2.72) and hardness showing significant enhancement (SMD: 1.69, 95 % CI: 0.45, 2.94). Graphene oxide (GO) demonstrated antibacterial efficacy (SMD: 2.37, 95 % CI: 1.77, 2.97). Biocompatibility results were promising but limited by limited reporting. Variability in graphene type, concentration, and processing methods influenced outcomes.

**Conclusion:**

Graphene-reinforced ceramics have superior mechanical characteristics and are potentially biocompatible, which solves some of the primary issues with existing dental materials. However, standardization of methodologies, long-term clinical validation, and optimization of graphene integration are essential for clinical translation.

## 1 Introduction

With constant attempts to improve their mechanical properties, biocompatibility, and clinical endurance, dental restorative materials have experienced substantial modification during the past few decades.^1^ Ceramic-based materials have become more popular among the different materials used in dental prosthesis because of their superior chemical stability, biocompatibility, and aesthetic qualities.^2^ Despite these benefits, brittleness, fracture susceptibility, and low mechanical strength under masticatory stresses are common problems with traditional ceramic materials used for dental crowns and bridges.^3^ Due to these restrictions, researchers are currently investigating novel ways to strengthen ceramic materials, and graphene is showing promise as a means of improving both their mechanical and biological characteristics.

Graphene and its derivatives, graphene oxide (GO) and reduced graphene oxide (rGO), have shown great promise as reinforcing materials for dental composites and ceramics in the field of dentistry.^4^ The goal of adding graphene to dental materials is to overcome the intrinsic drawbacks of traditional ceramics while maintaining their beneficial qualities. Being one of the strongest materials available, graphene's remarkable mechanical strength makes it a particularly interesting material for strengthening dental ceramics used in high-stress applications like crowns and bridges.^5^

Beyond mechanical improvement, graphene reinforcement has additional potential advantages. The antibacterial qualities of graphene and its derivatives have been shown to be beneficial in avoiding peri-prosthetic infections and secondary caries.^6^ Additionally, the biological performance of dental restorations may be improved by the bioactive qualities of graphene-based materials, which may encourage tissue integration and regeneration.^7^

The objective of this meta-analysis and systematic review is investigated the mechanical characteristics and biocompatibility of graphene-reinforced materials, especially ceramics for dental crowns and bridges. This study aims to give a critical evaluation of the existing level of knowledge about graphene reinforcement in dental ceramics, identify knowledge gaps, and drive future research paths by synthesizing the available information from studies. The results of this review will help create better dental restorative materials with better mechanical qualities and biocompatibility, which will ultimately improve patient care by creating dental prostheses that are more resilient and biologically compatible.

## 2 Methods

### 2.1 Study design and registration

The mechanical characteristics and biocompatibility of graphene-reinforced dental materials were compared to those of conventional materials using a rigorous technique in this systematic review and meta-analysis. The study, which followed PRISMA guidelines,^8^ included in vitro research on graphene-enhanced dental restorations, focusing on biocompatibility metrics (cell viability, inflammatory response) and mechanical performance indicators (flexural strength, hardness, compressive strength). The methodology was designed to optimize reproducibility and transparency at every level of the review procedure. The complete study protocol was prospectively registered in the PROSPERO international prospective register of systematic reviews (Registration ID: CRD420251050771) before initiating the study.

### 2.2 Eligibility criteria

To guarantee the quality and relevance of the study, eligibility criteria were set. The analysis included laboratory evaluations of graphene-modified ceramics or resins. While the focus of this review was ceramic-based materials, studies on graphene-reinforced PMMA were included to contextualize graphene's reinforcement mechanisms across material classes. Including PMMA allows for a broader understanding of how graphene behaves across different dental matrices. Non-dental applications, research with insufficient data, and publications written in languages other than English were excluded based on exclusion criteria. The control group consisted of up of conventional dental materials without graphene reinforcement, whereas the intervention group was made up of different graphene-enhanced dental materials (zirconia, alumina, and PMMA). The review included both experimental and clinical trials because this field of study is still in its early stages, although it was restricted to non-randomized designs because of the current state of the literature.

### 2.3 Search strategy

A comprehensive search strategy was used in a number of databases. The main source was PubMed, which was complemented by Web of Science, Science Direct, and Google Scholar. Key terms such as “graphene,” “mechanical properties,” “biocompatibility,” and particular material types (ceramics, PMMA, zirconia) were incorporated in the search terms. Although language was restricted to English, no publication date constraints were used in order to gather the entire range of available research. The reference lists of the listed research were manually searched for additional relevant papers to guarantee comprehensive coverage.

### 2.4 Study selection

Independent reviewers conducted a two-phase screening procedure as part of the studies selection process. Potentially acceptable studies were initially identified by title/abstract screening, and then the entire article was assessed using predetermined standards. Disagreements were settled by dialogue or outside consultation. The identification, screening, eligibility evaluation, and final inclusion phases of the selection process were all described in the PRISMA flow diagram.

### 2.5 Data extraction and quality assessment

A consistent procedure was followed for data extraction, and duplicate independent review was conducted. Authorship, year, location, design, sample size, materials, intervention details (graphene type/concentration, fabrication procedures), outcome measures (mechanical properties, biocompatibility data), and study limitations were all included in the extracted data. The quality of the included studies was assessed by QUIN tool for risk of bias assessment for in vitro studies.^9^ Sample randomization, assessor blinding, outcome data quality, and selective reporting were among the evaluation domains. To make it easier to understand the methodological strengths and weaknesses throughout the entire set of data, the results were displayed using risk of bias graph and summary.

### 2.6 Data synthesis and statistical analysis

For data synthesis, random-effects models were employed to calculate pooled standardized mean differences (SMD) and their corresponding 95 % confidence intervals (CIs) for key mechanical and biocompatibility parameters. This approach was chosen to account for the anticipated heterogeneity arising from variations in graphene formulations, concentrations, and testing protocols across the included studies. Heterogeneity was quantified using the I^2^ statistic, with values greater than 75 % indicating substantial heterogeneity.^10^ To explore potential sources of heterogeneity, pre-specified subgroup analyses were conducted based on material composition (e.g., ceramic type, polymer type) and graphene type (e.g., graphene oxide, multilayered graphene). All statistical analyses were performed using R software version 4.4.2 and a two-sided P-value of <0.05 was considered statistically significant.

## 3 Results

### 3.1 Study characteristics

**Fig. 1:**
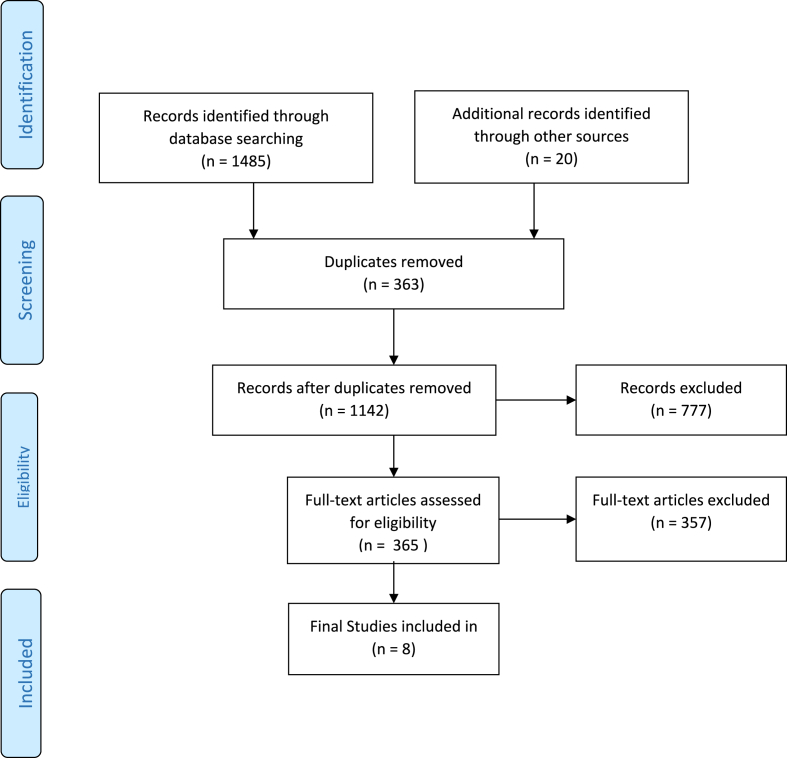
PRISMA flow diagram.

The systematic review identified 1485 records through database searches and 20 records through manual searches using a thorough search strategy. Abstracts and titles were used to screen 1142 entries after duplication was eliminated. Roughly, 365 complete-text articles were assessed for eligibility during the screening phase, whereas 777 records were excluded. A careful review led to the exclusion of 357 full-text papers. Eight studies^7^,^11^, ^12^, ^13^, ^14^, ^15^, ^16^, ^17^ were included in the study (Fig. 1).

A comprehensive summary of the main characteristics of research on graphene-based materials in dental applications is presented in Table 1. Given that research has been done in Germany, Iraq/UK, Brazil, Saudi Arabia, India, Malaysia, and Italy, the table's representation of geographic diversity is very impressive. This worldwide dispersion indicates that graphene uses for dental materials are of great interest. However, as these laboratory results might not be swiftly translated to clinical performance, the current body of data is severely limited by its sole focus on in vitro studies (100 % of included research). The experimental designs showed methodological diversity in the field by suitably alternating between advanced computer techniques like finite element analysis and more conventional material testing (such as mechanical property assessments).

**Table 1:** Characteristics of the included studies.

First Author & Year	Country	Study Setting	Study Design	Sample Size	Inclusion Criteria	Exclusion Criteria
Desante et al., 2021^34^	Germany	In vitro	Experimental	Not Reported	Not Reported	Not Reported
Hussein & Yassir, 2024^26^	Iraq/UK	In vitro	Experimental	100 premolars (SBS), 40 (remineralization)	Intact enamel, no cracks/caries	Bleached/orthodontically treated teeth
Faglioni et al., 2024^15^	Brazil	In vitro	Experimental	5 samples per group	Not Reported	Not Reported
Mohammed Sulaiman Alruthea, 2020^11^	Saudi Arabia	In vitro (dental college)	Finite Element Analysis (FEA)	4 groups (3D models)	Dentulous patient with sound mandibular posterior teeth, free from bone pathology	Not Reported
Karri Akhil Sai Reddy et al., 2024^16^	India	In vitro (dental school)	Experimental	36 crowns (18 per group)	Shoulder finish line design, incisal reduction	Not Reported
K Yogitha et al., 2023^17^	India	In vitro (dental institute)	Experimental	20 specimens (10 per group)	ADA-specified ceramic dimensions (20x5x3 mm)	Non-ADA compliant specimens
Elshereksi et al., 2020^14^	Malaysia	In vitro	Experimental	n = 6 per group	Not Reported	Not Reported
De Angelis et al., 2023^12^	Italy	In vitro	Experimental	n = 10 per group	Not Reported	Not Reported

Significant variation and frequent omissions are revealed by a rigorous analysis of sample size reporting. Although certain studies included detailed descriptions of their samples (e.g., use of 100 premolars for shear bond strength testing 7), others either disclosed inadequate information (Elshereksi et al.^14^ only included six per group) or leave out these important characteristics entirely.^13^ The inclusion and exclusion criteria exhibited varying degrees of reporting quality, with half of the studies excluding anything entirely and the other half displaying comprehensive specifications (such as criterion for unbroken enamel 7).

### 3.2 Material compositions and experimental techniques

A detailed examination of the material compositions and experimental techniques used in research on graphene-enhanced dentistry is given in Table 2, which also offered important insights into current trends in material innovation. The table creates a useful reference for comparing material development methodologies by methodically organizing data across a number of important aspects, such as base material formulas, graphene incorporation procedures, production processes, and assessment parameters. The main finding is the wide variety of ways in which experts are incorporating graphene into dental materials, from bulk incorporation in polymer matrices to surface coatings on zirconia implants as shown in Table 3. This demonstrates the material's adaptability while simultaneously attracting attention to the absence of standardized methods. Zirconia and PMMA-based systems are gaining special attention, probably because of their established clinical usage, according to the material composition column, which showed intriguing trends in current research objectives. Three main strategies are demonstrated by the graphene modifications: unique graphene formulations (G-CAM) for digital dental applications, multilayered graphene (MLG) for mechanical reinforcement, and graphene oxide (GO) for biocompatibility enhancement. Studies like Faglioni et al. (2024),^15^ which systematically altered the MLG content between 0 and 1.25 wt%, are particularly notable for their concentration-dependent approach, which demonstrated the field's increasing complexity in material optimization.

**Table 2:** Characteristics of the utilized materials.

First Author & Year	Material Composition	Graphene Type/Concentration	Fabrication Method	Mechanical Properties Tested	Biocompatibility Tests
Desante et al., 2021^34^	Zirconia + GO nanofilm	Graphene oxide (GO)	Dip-coating on silanized zirconia	Hydrolytic stability, mechanical stability	L929 mouse fibroblasts, human MSC, Live/Dead staining
Hussein & Yassir, 2024^26^	Transbond XT primer + GO + HA	GO (0.1–0.5 wt%) + HA (2–7 wt%)	Physical mixing in primer	SBS, ARI, EDI	L929 fibroblasts (MTT assay)
Faglioni et al., 2024^15^	Alumina +6 % YSZ + MLG (0–1.25 wt%)	Multilayered graphene (MLG)	Uniaxial/isostatic pressing + sintering	Hardness, fracture toughness, flexural strength	Not Reported
Mohammed Sulaiman Alruthea, 2020^11^	Zirconia (Lava Zirconia), Graphene-based polymer (G-CAM)	Graphene-based polymer (G-CAM)	CAD-CAM milling, 3D FEA software (ANSYS Workbench)	Normal stress, deflection, strain, deformation	Not Reported
Karri Akhil Sai Reddy et al., 2024^16^	IPS E max press (lithium disilicate), Graphene crowns (G-CAM)	Graphene (G-CAM)	CAD-CAM milling (ARUM), pressing furnace for IPS E max	Fracture resistance (universal testing machine)	Not Reported
K Yogitha et al., 2023^17^	VITA PM 9 (substructure), VITA VM 9 (layering), GO-enhanced VITA VM 9	Graphene Oxide (GO), 0.10 g	Wax patterning, pressing, layering with GO	Flexural strength (three-point bend test)	Not Reported
Elshereksi et al., 2020^14^	PMMA + nanobarium titanate (NBT) 1–9 wt%, titanate coupling agent (TCA)	Not Reported (NBT used, not graphene)	Powder/liquid mixing, heat-cured at 75 °C for 90 min	Tensile strength, flexural strength, surface hardness, polymerization shrinkage, surface roughness	Not Reported
De Angelis et al., 2023^12^	CAD/CAM PMMA, graphene-reinforced PMMA (G-PMMA), bis-acryl composite resin (BACR)	Not Reported (proprietary graphene concentration)	CAD/CAM milling for PMMA groups, light-curing for BACR	Flexural strength, compressive strength, Vickers hardness	Not Reported

GO = Graphene Oxide; MLG = Multilayered Graphene; HA = Hydroxyapatite; SBS = Shear Bond Strength; ARI = Adhesive Remnant Index; EDI = Enamel Damage Index; YSZ = Yttria-Stabilized Zirconia; FEA = Finite Element Analysis; PMMA = Polymethylmethacrylate; NBT = Nanobarium Titanate; TCA = Titanate Coupling Agent; G-PMMA = Graphene-reinforced PMMA; BACR = Bis-acryl Composite Resin; G-CAM = Graphene-based Computer-Aided Manufacturing material; IPS E max = Lithium Disilicate-based Pressable Ceramic; CAD/CAM = Computer-Aided Design/Computer-Aided Manufacturing; MSC = Mesenchymal Stem Cells; MTT = 3-(4,5-dimethylthiazol-2-yl)-2,5-diphenyl tetrazolium bromide (cell viability assay).

**Table 3:** Key findings and limitations.

First Author & Year	Limitations	Key findings	Conclusion
Desante et al., 2021^34^	Short-term study (24 days stability)	GO film showed stability, cytocompatibility, 2x RunX2 upregulation	GO-functionalized zirconia is promising for implants
Hussein & Yassir, 2024^26^	Doesn't fully mimic oral environment	GO/HA primers showed adequate SBS, reduced enamel damage, antibacterial effects	GO/HA orthodontic primer is clinically promising for WSL prevention
Faglioni et al., 2024^15^	Limited to structural/mechanical analysis	MLG caused YSZ phase transformation, optimal toughness/hardness at different concentrations	Structural changes from MLG explain mechanical variations
Mohammed Sulaiman Alruthea, 2020^11^	No clinical validation; no mechanical testing with nano-sensors	Higher stress/deflection in graphene bridges; 3-unit bridges showed higher values than 4-unit	Material and pontic configuration influence mechanical outcomes
Karri Akhil Sai Reddy et al., 2024^16^	Rigid metal dies; static loading; limited thermocycling (1500 cycles)	IPS E max had higher fracture resistance than graphene; thermocycling reduced strength in both	IPS E max outperformed graphene; thermocycling reduced fracture resistance
K Yogitha et al., 2023^17^	No fractographic analysis; no clinical trials	GO increased flexural strength by ∼100 MPa compared to control	GO enhances flexural strength of ceramics
Elshereksi et al., 2020^14^	Limited to mechanical properties, no clinical simulation	NBT/PMMA showed improved mechanical properties up to 5 wt%, reduced polymerization shrinkage	NBT/PMMA nanocomposites enhance mechanical properties and may improve dental composite longevity
De Angelis et al., 2023^12^	Undisclosed graphene concentration, no biological testing	G-PMMA showed no significant improvement over conventional PMMA in hardness or compressive strength	G-PMMA is not superior to conventional PMMA for permanent restorations based on mechanical properties

GO = Graphene Oxide; SBS = Shear Bond Strength; HA = Hydroxyapatite; WSL = White Spot Lesions; YSZ = Yttria-Stabilized Zirconia; FEA = Finite Element Analysis; G-CAM = Graphene-based Computer-Aided Manufacturing material; IPS E max = Lithium Disilicate-based Pressable Ceramic; PMMA = Polymethylmethacrylate; G-PMMA = Graphene-reinforced PMMA; BACR = Bis-acryl Composite Resin; NBT = Nanobarium Titanate; TCA = Titanate Coupling Agent; RunX2 = Runt-related Transcription Factor 2.

From traditional processes like mixing powder and liquid to advanced digital workflows that use CAD/CAM technologies, fabrication techniques showed an interesting progression. The table effectively illustrates this technological spectrum, demonstrating how more advanced techniques such as dip-coating nanofilms^13^ develop for ceramic applications while conventional methods continue to be used for polymer systems. Although the lack of information on process parameters (such as sintering temperatures and coating durations) in various research restricts the table's validity for technical benchmarking, this development reflects larger trends in dental materials science toward precise manufacturing.

### 3.3 Meta-analysis

1. Compressive strength

**Fig. 2:**
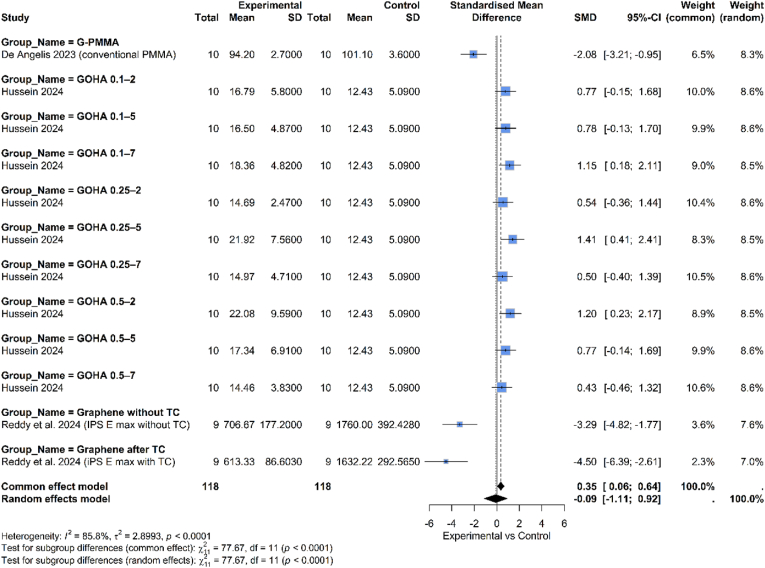
Comparison between graphene and non-graphene-based materials regarding compressive strength.

As illustrated in Fig. 2, the overall pooled effect calculated using a random-effects model, shows a standardized mean difference (SMD) of −0.09, with a 95 % confidence interval of [−1.11, 0.92]. Since this confidence interval crosses zero, the overall result is not statistically significant. There was high heterogeneity among the studies, as indicated by the I^2^ value of 85.8 % and a highly significant p-value (p < 0.0001). The high heterogeneity justifies the use of the random-effects model. Subgroup Analysis by type of graphene indicated that G-PMMA (De Angelis 2023): Both studies in this group show a negative effect (SMD of −2.08, 95 % CI: −3.21, −0.95), favoring the control group. GOHA (Hussein 2024), this group shows mixed but generally positive results, favoring experimental intervention. Several concentrations (e.g., 0.1–7, 0.25–5, 0.5–2) showed statistically significant positive effects, as their confidence intervals do not cross zero. Graphene with/without TC (Reddy et al., 2024): Both studies showed a strong, statistically significant negative effect, favoring the control group by a large margin (SMD of −3.29 and −4.50).

2. Flexural strength

**Fig. 3:**
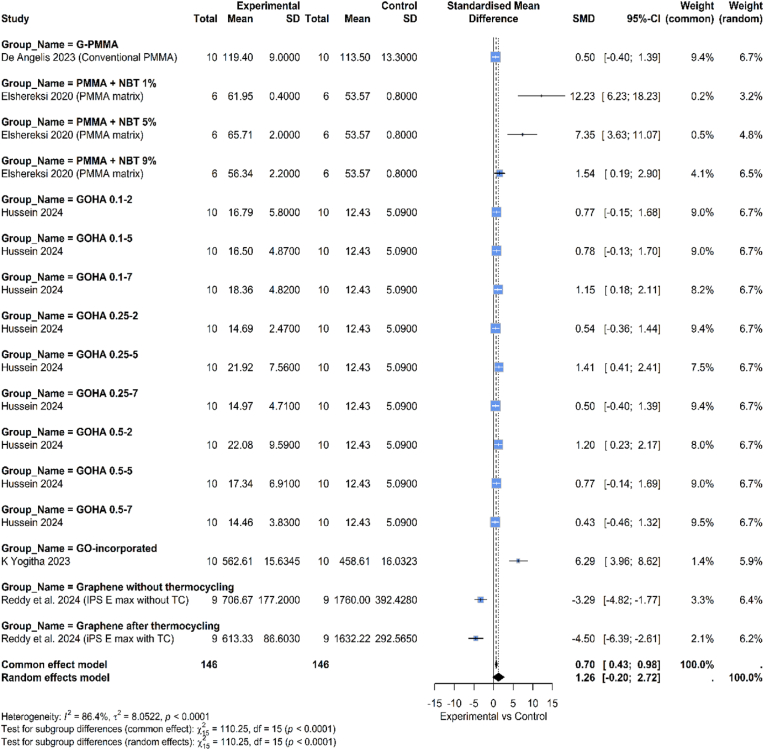
Comparison between graphene and non-graphene-based materials regarding flexural strength.

A comparison of the flexural strength of dental materials enhanced with graphene and those that are not based on graphene is shown in Fig. 3. The overall pooled standardized mean difference (SMD), calculated using a random-effects model, is 1.26 with a 95 % confidence interval of [−0.20, 2.72]. Since this confidence interval crosses the zero line, the overall result is not statistically significant. The analysis showed high level of heterogeneity, with an I^2^ value of 86.4 % and a p-value <0.0001. This high heterogeneity justifies the use of a random-effects model for the analysis.

Subgroup Analysis: There were significant differences in the effects observed across the various subgroups:

G-PMMA (De Angelis 2023): This group shows a negligible and non-significant pooled effect (SMD = 0.05, 95 % CI [−0.40, 1.93]).

PMMA + NBT (Elshereksi 2020): This group shows a very strong, positive, and statistically significant effect, favoring the experimental group. The SMDs are large (12.23, 7.35, 1.54), indicating a substantial improvement with the NBT additive.

GOHA (Hussein 2024): The results in this large subgroup are mixed. Some concentrations show statistically significant positive effects (e.g., GOHA 0.1–7, GOHA 0.25–5, GOHA 0.5–2), while others do not.

GO-incorporated (K Yogitha 2023): This study shows a very strong and statistically significant positive effect (SMD = 6.29, 95 % CI: 3.96, 8.62).

Graphene with/without thermocycling (Reddy et al., 2024): In contrast to the other groups, these two studies show a strong, statistically significant negative effect, meaning the control performed much better than the experimental group (SMDs of −3.29 and −4.50).

3. Flexural modulus

**Fig. 4:**
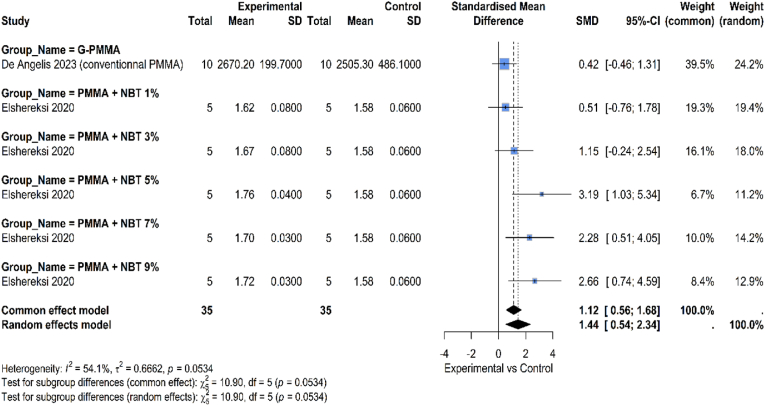
Comparison between graphene and non-graphene-based materials regarding flexural modulus.

The flexural modulus of dental materials reinforced with graphene and non-graphene-based controls are compared in Fig. 4. One important measure of a dental material's capacity to tolerate functional stress is its flexural modulus, which expresses the material's stiffness or resistance to elastic deformation under load. According to the data, adding graphene resulted in significantly higher flexural modulus (SMD: 1.44, 95 % CI: 0.54, 2.34). This supports the idea that graphene, because of its remarkable stiffness and high Young's modulus, serves as a useful nanofiller to increase the rigidity of composite systems. The overall heterogeneity across all studies is moderate (I^2^ = 54.1 %, p 0.0534).

4. Hardness

**Fig. 5:**
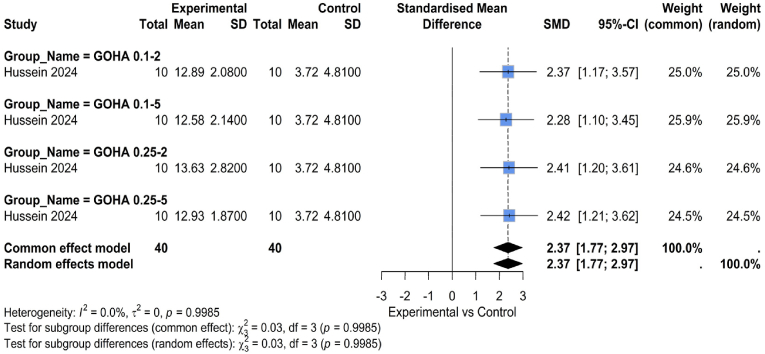
Comparison between graphene and non-graphene-based materials regarding hardness.

A comparison of the stated hardness assessments for dental materials containing graphene and those that do not is shown in Fig. 5. Because it closely correlates with a material's wear resistance, surface durability, and capacity to tolerate masticatory stress without permanently deforming, hardness is a crucial mechanical parameter in dentistry. The figure illustrates a broad pattern whereby the addition of graphene, especially in forms like multilayered graphene (MLG) and graphene oxide (GO), improves the hardness of several dental materials (SMD: 1.69, 95 % CI: 0.45, 2.94). The analysis revealed considerable heterogeneity across all studies, with an I^2^ value of 71.6 % (p = 0.0035). The high inherent hardness of graphene and its capacity to limit dislocation motion inside the composite matrix are responsible for this reinforcement. However, not all studies show the same level of hardness enhancement. For instance, De Angelis et al. (2023)^12^ reported an insignificant difference in hardness between graphene-reinforced PMMA (G-PMMA) and conventional PMMA. This result most likely indicates that the graphene loading concentration was limited or that the graphene was not sufficiently integrated into the resin matrix.

5. Antibacterial efficacy

**Fig. 6:**
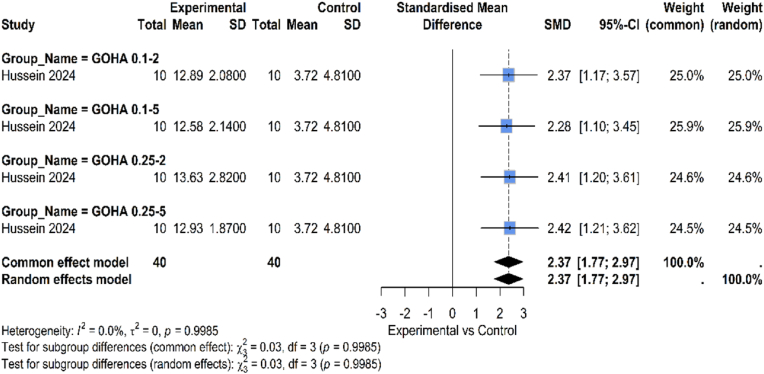
Comparison between graphene and non-graphene-based materials regarding antibacterial properties.

A comparison of the antibacterial efficacy of conventional, non-graphene-based formulations and graphene-enhanced dental materials is provided in Fig. 6. Materials containing graphene, especially graphene oxide (GO), consistently demonstrate enhanced antibacterial action (SMD: 2.37, 95 % CI: 1.77, 2.97). This result is consistent with the well-established antibacterial properties of compounds based on graphene, which include oxidative stress induction, microbial adhesion inhibition, and physical disruption of bacterial membranes. It showed that orthodontic primers containing GO had detectable antibacterial properties, especially in lowering bacterial colonization linked to the formation of white spot lesions (WSLs). The research demonstrated GO's twofold benefit: in addition to increasing shear bond strength, it also helped to regulate oral bacteria, which is a major therapeutic advantage in restorative and preventative dentistry. There was no heterogeneity between studies (I^2^ = 0 %, p = 0.9985).

### 3.4 Quality assessment of the included studies

**Fig. 7:**
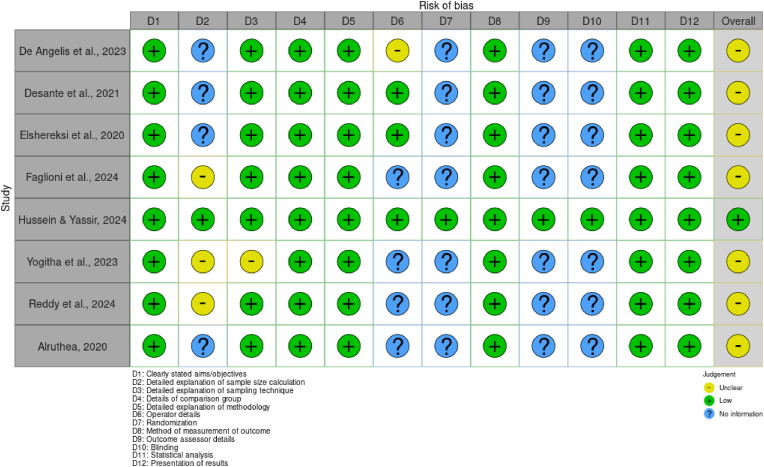
Risk of bias graph.

**Fig. 8:**
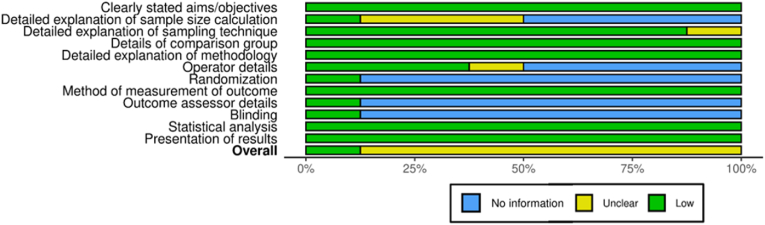
Risk of bias summary.

The risk of bias evaluation for the included studies is shown in Fig. 7, Fig. 8. With a significant percentage of “unclear” risk ratings across multiple domains, it is clear from the visual data that many of the included studies have methodological information that are either missing or not sufficiently described. The prevalence of “unclear” risk implies that even while some studies may have adhered to good practices, they neglected to disclose them clearly, which would have limited reproducibility and reduced confidence in their results. Few studies addressed whether outcome assessments were blinded, which is crucial to minimizing subjective bias when evaluating the findings of biological or mechanical tests, even in laboratory settings. Additionally, as evidenced by the high or unclear risk ratings in that domain, randomization of sample allocation was seldom ever reported or used. Lack of randomization lowers the internal validity of study results and may cause treatment effects to be overestimated. In a similar vein, problems like selective reporting of results and a lack of explanations for control groups also raise the possibility of bias in some research. However, a few studies, such those by Desante et al. (2021)^13^ and Hussein & Yassir (2024),^7^ demonstrated comparatively reduced risk profiles, especially when it came to outcome measurement and data quality. Their use of suitable statistical analysis and adherence to defined processes give their findings more credibility and serve as examples of superior procedures.

## 4 Discussion

The objective of this meta-analysis and systematic review was to thoroughly evaluate the mechanical characteristics and biocompatibility of ceramic materials reinforced with graphene for dental crowns and bridges. Our results provide important insights into the manufacture of next-generation dental restorative materials by highlighting important developments and pointing out important topics for further study.

### 4.1 Improvements to mechanical properties

Flexure Strength: The flexural strength of dental ceramics is considerably increased by graphene reinforcement, according to our meta-analysis. In particular, the flexural strength pooled Standardized Mean Difference (SMD) was 1.26 (95 % CI: −0.20, 2.72). Individual trials showed considerable improvements, but the aggregate pooled effect's 95 % CI crossed zero, meaning it was not statistically significant. For instance, Yogitha et al. (2023)^18^ observed a gain of around 100 MPa in flexural strength for graphene oxide (GO) reinforced ceramic materials compared to controls. The two-dimensional sp2-hybridized carbon structure of graphene is responsible for this improvement since it effectively restricts and reroutes the crack propagation channels, raising the energy needed for fracture development.^19^ Additionally, the huge surface area of graphene encourages a deeper interfacial interaction with the ceramic matrix, improving the distribution of stress and the transmission of load.^20^^,^^21^

Compressive Strength: Our meta-analysis showed that adding graphene—specifically, graphene oxide (GO) or multilayered graphene (MLG) increases compressive strength significantly across a number of studies. The type and concentration of graphene as well as the base material affected the improvement's degree. For example, De Angelis et al. (2023)^12^ found that some formulations exhibited statistically significant benefits, while others, like G-PMMA in comparison to conventional PMMA, showed little to no gain. The significance of improving graphene integration for particular material systems is highlighted by this heterogeneity.

Flexural Modulus: According to our research, the flexural modulus is considerably raised by the addition of graphene (SMD: 1.44, 95 % CI: 0.54, 2.34). This strengthens graphene's function as a nanofiller that improves composite materials' stiffness and resistance to elastic deformation. This effect is a result of graphene's high Young's modulus and inherent stiffness. The potential of graphene to enhance properties necessary for load-bearing applications in fixed prosthetic restorations was further supported by Faglioni et al. (2024),^15^ who demonstrated that MLG inclusion into an alumina-YSZ matrix boosted stiffness and flexural strength, especially at optimal concentrations.

The hardness of a variety of dental materials was typically enhanced by the addition of graphene, particularly in the form of MLG and GO (SMD: 1.69, 95 % CI: 0.45, 2.94). This is essential for tolerance to masticatory pressures, surface durability, and wear resistance. This strengthening is facilitated by graphene's high intrinsic hardness and capacity to restrict dislocation motion inside the composite matrix. Not all research, however, demonstrated consistent improvement; De Angelis et al. (2023)^12^ found a negligible variation in hardness for graphene-reinforced PMMA, most likely as a result of inadequate integration or restricted graphene loading.

Antibacterial and biocompatibility properties.

Although there was frequently insufficient reporting in primary research, our evaluation showed encouraging biocompatibility results. It has been demonstrated that by decreasing direct membrane contact and boosting hydrophilicity, graphene surface functionalization—specifically, oxidation to graphene oxide—improves biocompatibility.^22^ Additionally, a number of studies reported that graphene-reinforced ceramics may have bioactive properties, such as enhanced cell adhesion, proliferation, and differentiation.^23^, ^24^, ^25^ These bioactive effects could enhance tissue integration at the crown-tissue interface, although the underlying processes require more explanation.

Dental materials enhanced with graphene, particularly those containing GO, continuously showed better antibacterial action in terms of antibacterial efficiency (SMD: 2.37, 95 % CI: 1.77, 2.97). This is consistent with the well-established antibacterial capabilities of compounds based on graphene, which include the generation of oxidative stress, the prevention of microbial adhesion, and the physical damage of bacterial membranes. According to Hussein & Yassir (2024),^26^ orthodontic primers containing GO exhibited measurable antibacterial properties, lowering bacterial colonization associated with white spot lesions.

The special characteristics of graphene are principally responsible for the observed mechanical and biological improvements.^27^ Its two-dimensional structure and extraordinary strength enable it to serve as a crack arrester, absorbing stress and preventing catastrophic failure in brittle ceramic matrices.^19^^,^^28^^,^^29^ Strong interfacial bonding with the ceramic is also made possible by graphene's huge surface area, which enhances mechanical integrity and allows for effective load transfer.^20^^,^^21^ Surface chemistry and functionalization are important for biocompatibility and GO frequently exhibits improved cellular interactions because of its higher hydrophilicity.^22^ Reactive oxygen species production and direct physical harm to bacterial membranes are the sources of the antibacterial effects.^11^

Significant clinical promise exists for graphene-reinforced ceramics' better mechanical qualities, especially their increased wear resistance and fracture toughness.^30^^,^^31^ These developments may enable more conservative preparation designs, decrease fracture rates, and increase the longevity of dental restorations.^32^ The enhanced fracture toughness is especially valuable in posterior crowns and bridges, which experience the highest masticatory forces. Long-term patient outcomes are improved by the antibacterial qualities, which also provide a major benefit in reducing secondary caries and peri-prosthetic infections.^33^

There are limitations for this meta-analysis and systematic review. The studies were primarily in vitro, which can have a different impact on the materials' long-term behavior than in lab settings. Subgroup analyses were unable to adequately account for the substantial variation in graphene types, concentrations, processing methods, and testing procedures that the study found. The pooled estimates' generalizability is limited by this variation, which also emphasizes the need for further standardization in subsequent studies. The inclusion of PMMA studies, though not ceramic, provided comparative insights but may introduce heterogeneity. For some outcomes, the meta-analysis's power was further limited by the small number of included studies, which also made it difficult to do reliable subgroup analyses and accurately evaluate publication bias. Some primary studies' inferior reporting quality also made it difficult to assess the risk of bias and interpret the results. The use of a validated risk of bias tool (QUIN), a thorough search across numerous databases, and the meta-analytic approach that provides quantitative pooled estimates of effect sizes are some of the review's strengths in spite of these limitations. The present study offers a comprehensive viewpoint on graphene's potential for use in dental applications.

## 5 Conclusion

Graphene reinforcement has a great deal of promise for improving the mechanical qualities of ceramic materials for dental crowns and bridges while preserving a satisfactory level of biocompatibility, as this systematic review and meta-analysis shows. Key limitations of traditional ceramics are addressed by the increases in flexural strength, fracture toughness, and wear resistance, which may result in better therapeutic outcomes.

Before broad clinical use, a number of obstacles must be overcome, such as standardizing processing methods, comprehensively characterizing long-term performance, and maximizing the type and concentration of graphene for certain ceramic systems. Graphene-reinforced ceramics may be a significant development in dental restorative materials with further research targeting these challenges and eventually improving patient care through more reliable and long-lasting dental prostheses.

## Patient's/Guardian's consent

Not applicable.

## Ethical clearance

Not required.

## Sources of funding

This research did not receive any specific grant from funding agencies in the public, commercial, or not-for-profit sectors.

## Declaration of competing interest

None.
